# Characteristics of Water Transport of Membrane Electrolyte over Selected Temperature for Proton Exchange Membrane Fuel Cell

**DOI:** 10.3390/polym14152972

**Published:** 2022-07-22

**Authors:** Ngoc Van Trinh, Xuan Linh Nguyen, Younghyeon Kim, Sangseok Yu

**Affiliations:** 1 Graduate School, Chungnam National University, Daejeon 34134, Korea; 201950184@o.cnu.ac.kr (N.V.T.); linhnx@o.cnu.ac.kr (X.L.N.); viny9198@cnu.ac.kr (Y.K.); 2 Department of Mechanical Engineering, Chungnam National University, Daejeon 34134, Korea

**Keywords:** diffusivity, convection mass transfer, proton-exchange membrane fuel cell, vapor transport, diffusion mechanism, performance and durability

## Abstract

The water contents at both the anode and cathode of PEMFCs depend on the water-transport mechanism at the membrane. The humidity at the outside layers of the membrane determines the diffusion of water through it. The operating temperatures and pressures regulate the humidity conditions in the system. Because these parameters are nonlinear, the water-transport mechanism is analyzed via the difference in the water concentration on each side of the membrane. In this work, an experimental configuration is designed to investigate the diffusion mechanism of water through the membrane. A flat membrane module is tested in an isothermal test chamber to test the influence of temperature on the water-absorption and -transport characteristics of Nafion 117 and Nafion 211 membranes. A parametric study is conducted to test the water-transport mechanism at an operating pressure of 1 bar; temperatures of 30 °C, 50 °C, 70 °C and 90 °C; and a relative humidity ranging from 10% to 100%. The results indicate that the water content of Nafion 211 is higher than that of Nafion 117. The water content and diffusion coefficient are proportional to the operating temperature. In addition, the diffusion coefficient reaches its peak at conditions of 1 bar, 100% humidity, and 90 °C for both membrane types.

## 1. Introduction

The exploitation of fossil fuels in power generation and transportation has led to devastation of the global environment, and carbon dioxide emissions have increased tenfold in the last five decades. Thus, climate change and global warming have become significant problems in the twenty-first century. There is an urgent need for alternative sources of renewable energy. However, the available renewable energy sources are unstable which makes it difficult to maintain the system continuously. To tackle this issue, the employment of energy storage systems combined with renewable energy may greatly improve the utilization rate and stability of renewable energy [1]. The fuel cell is a potential candidate with high efficiency and reliable performance. In particular, proton-exchange membrane fuel cells (PEMFCs) could be applied in many areas. In addition, their advantage over other FC techniques is that their use of an ion-conductive and/or optimized porous membrane is believed to improve the cycling stability of the battery by suppressing dendrite formations [2]. The stack, a series connection of many unit cells, is the core of this fuel cell system. A redox reaction occurs between the electrodes to generate electricity, heat, and water. Depletion and excess water inside the stack both result in drastic declines in performance and durability. Flooding in the stack interferes with the oxygen supply from the air electrode, whereas drying interferes with hydrogen transport in the electrolytic membrane. Therefore, the proton-exchange membrane (PEM) is essential to the system’s performance.

Nafion membranes are among the most commonly used PEMs, and there have been many studies on their use in water-transport mechanisms [3,4,5,6,7,8,9,10,11,12,13,14,15]. Zawodzinski and Springer evaluated the performance of the Nafion 117 membrane by determining the water content and corresponding diffusion coefficient at 30 °C using nuclear magnetic resonance (NMR), which shows the water content of the membrane according to the relative humidity at the same operating temperature [3,4]. Maldonado compared the physical properties of Nafion 115 and Nafion 212. The results indicated that the water uptake, proton conductivity, and water self-diffusion coefficient decline when the drying temperature increases [16]. Daniel J. Burnett determined the water sorption and diffusion properties of three Nafion membranes, N-117, N-112, and NR-112, using dynamic gravimetric vapor sorption instruments. The results show that when the temperature increases, the diffusion coefficient also rises and peaks at an intermediate humidity level. Additionally, the water absorption depends on the thickness of the membrane; the thicker N-117 has a lower percentage uptake than the thinner N-112 [17]. Qiongjuan Duan presented liquid water-transport through the Nafion membrane using convective water-transport instead of diffusion. Convective flow from the applied hydrostatic water pressure is greater than the estimated diffusive water flux associated with the water activity gradient [18]. K. Chadha evaluated the efficiency of water diffusion through N-211 using the water-balance calculation method. A value between 1 × 10^−^^7^ and 4 × 10^−^^7^ cm^2^/s was reported, which indicated that the water-balance method can be applied easily to measure an effective membrane water-diffusion coefficient. Furthermore, this method could also be used to calculate the water production and distribution in the fuel cell stack [19].

Although there are extensive studies of the physical properties of Nafion membranes, such as N-117, N112, and N115, it is still necessary to investigate the water-transport performance of newly developed membrane electrolytes. In this study, a newly designed isothermal test jig is used to understand the water-transport process of a membrane electrolyte and measure the effective water-transport through it. The water-transport characteristics of NR211 are then compared with those of Nafion 117 at a temperature of 30 °C. In addition, the water-transport performance is investigated at an operating pressure of 1 bar and temperatures of 30 °C, 50 °C, 70 °C and 90 °C. At each operating temperature, the relative humidity is maintained from 10% to 100%. The water-transport characteristics are then used to calculate the diffusion coefficients of the membrane electrolyte, which depends on the temperature and vapor concentration.

## 2. Experimental Approach

### 2.1. Experimental Configuration

**Figure 1:**
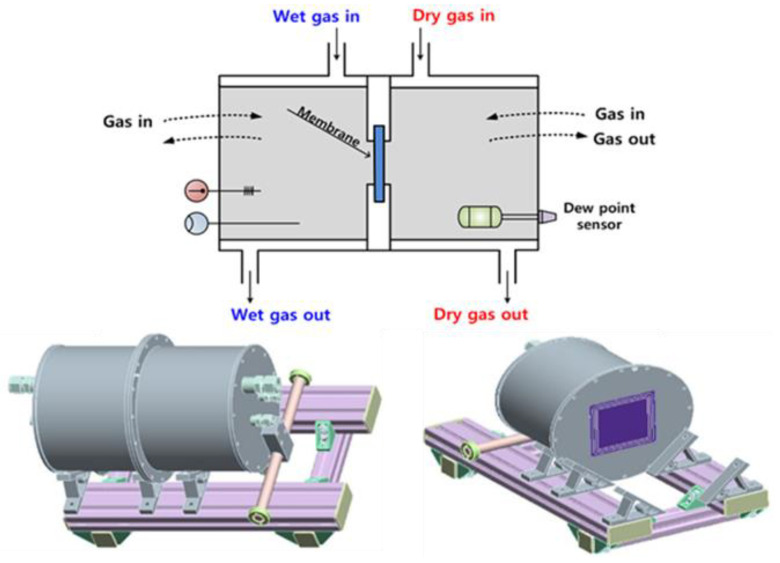
Design of the isothermal test jig.

Figure 1 shows the design of test jig to study water-transport through the electrolytic membrane. It has a large chamber divided into two equally spaced sections with the membrane in the middle. The test jig has four gates: wet gas in, dry gas in, wet gas out, and dry gas out. Moist air flows into the wet side, and the dry air is poured into the dry side. The membrane is placed in the middle of the test jig, and the system is sealed with two round metal plates to seal the system and prevent air leakage. Each metal plate is designed with a rectangular hole precisely corresponding to the required experimental area for the membrane. The outside of shell is heated uniformly, and thick insulation is also attached. The temperatures of the test jig, dry inlet/outlet stream, and wet inlet/outlet stream are maintained at the same level to achieve isothermal conditions.

The specifications of Nafion 211 and Nafion 117 are enumerated in Table 1. The experimental area is of 49 cm^2^ is used to test the diffusion mechanism of the system. In addition, to ensure reliable system performance, the membrane must be dried out for 24 h before the experimental procedure is performed. A vacuum dryer with the specifications detailed in Table 2 is used to dry the membrane.

**Figure 2:**
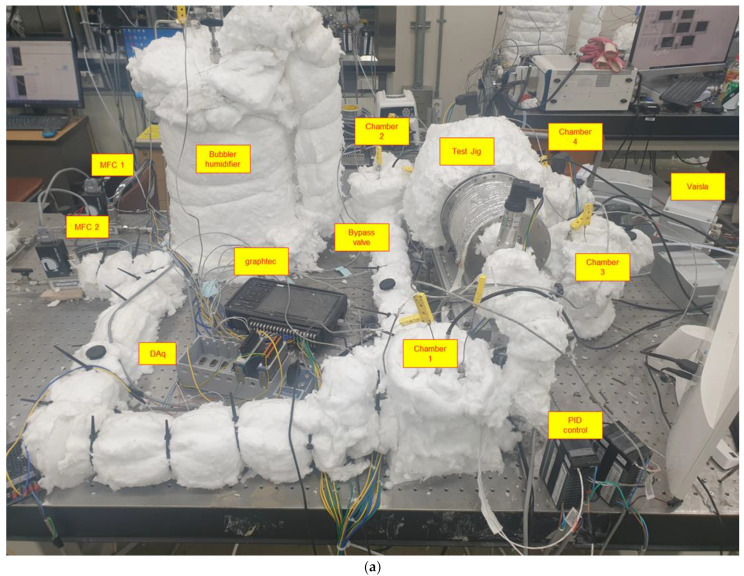

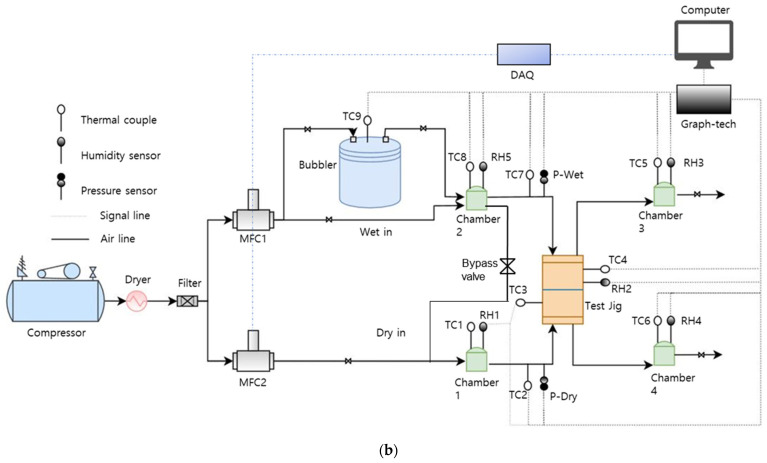
(**a**) Experimental apparatus and (**b**) schematic diagram.

Figure 2 is a photo and schematic diagram of experimental apparatus used measure the water-transport rate of the membrane. The total system mainly comprises an air-supply system, bubbler humidifier, test jig, air-stabilization chamber, and control and measurement system. Before air is supplied into the system, it is delivered through a compressor, dryer, and filter for desiccation and refinement. Because the dehumidification process produces fully desiccated air, the experimental uncertainty is negligible. Next, the air is separated into two paths. One goes directly to the test jig, and the other is mixed with water vapor bubbler humidifier to meet the specified relative humidity before flowing into the test jig. The air each side is controlled by a mass flow controller (MFC) to eliminate disturbance to the system. The humidifier was controlled by adjusting the water temperature using the heating rod inside the humidifier. The heating rod inside the humidifier is used with a 2.5 kW heat capacity, according to the heat capacity of the water. A line heater is applied to both sides of the test jig to maintain the temperature and prevent condensation. In addition, insulation (ceramic fiber) was applied to the exposed portions of the metal over the whole system. K-type thermocouples and pressure sensors were used to measure the temperature and pressure. Humidity sensors (Vaisala, HMT 337) were used to measure the relative humidity in Chamber 1–4 and on the wet side of the test jig. In Chamber 3, the temperature was approximately 5 °C higher to prevent the condensation. The temperature was controlled using an automatically tuned proportional-integral-differential (PID) value from the temperature controller (UT-32A). The PID controller was calibrated for about 12 h and tested several times to test the system’s reliability before being applied to the actual temperature controller. The back pressure of the airflow controlled the operating pressure of the whole system.

**Figure 3:**
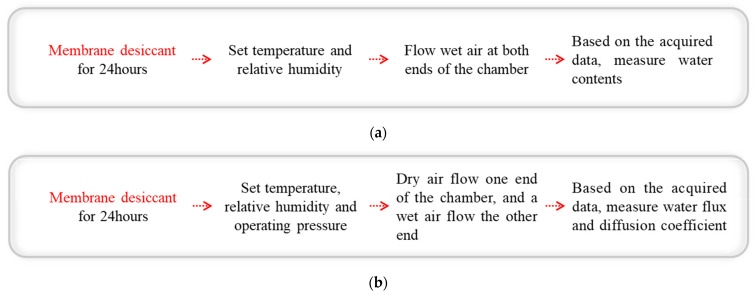
Experimental procedure. Measurement of (**a**) water content and (**b**) water-transport.

Figure 3a demonstrates the experimental procedure to measure the water content. Because the initial water content of the Nafion membrane should be set to 0 to ensure accurate measurement of the water content and water-diffusion coefficient, the Nafion membrane is vacuum dried at 25 °C and 1 bar for 24 h before each experiment. The bypass valve from Chamber 2 to the dry side of the test jig is opened and MFC-2 turns off when the water content is measured. After 2 h under individual experimental conditions, and the membrane is fully hydrated. An electronic scale (GX-200) is used to measure the mass of water in the Nafion^®^ membrane. The experimental conditions are presented in Table 3.

Figure 3b describes the water-transport measurement process. The experimental data are captured when the system reaches the steady-state condition. The bypass valve, located behind Chamber 2, controls the relative humidity of the inlet wet side by mixing the dry air with vapor from the bubbler humidifier. The relative humidity for the experiment ranges from 10% to 100%. A Graphtec data logger is used to record the experimental data. The temperature, humidity, and pressure at each point were sampled every second and then averaged. Each experiment was conducted at least twice to ensure reliability. The errors between the repeated experiments were less than 3% in terms of each measured parameter.

### 2.2. Mathematical Background

**Figure 4:**
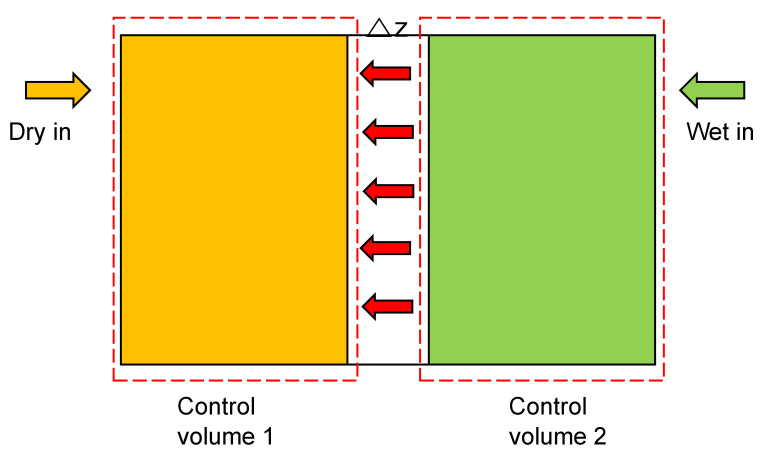
Control volume analysis of water-vapor transport.

Figure 4 presents the control volume analysis of water vapor transport. The experimental configuration is suitable for the mixture of vapor and air on the wet side to flow uniformly into the test jig. Because of the PID controller and heated system, the temperature inside the test jig is maintained constantly. In addition, the diameter of the test jig is much larger than the tube, and the fluid flow velocity inside the Test Jig is small. As a result, it is suitable to consider that the diffusion mechanism can be considered the dominant water-transport mechanism through the membrane. The following assumptions should be considered:

1. The experiments are conducted under isothermal steady-state conditions for both air streams.
2. There are no chemical reactions either side of the test jig.
3. No condensation occurs inside or outside the test jig. The wet air is considered an ideal gas.
4. The membrane is a stationary, nonreacting medium of uniform total molar concentration.

The water content of the membrane is determined by the mass difference between the dry and wet membranes. A precise electronic scale is used to measure the total membrane weight before and after the experiment. The water content of the electrolytic membrane is defined as the ratio of the concentration of sulfonic acid groups to the concentration of water. Therefore, the water content is determined as follows:

(1) $$\lambda =\frac{C_{H_{2}O}}{C_{{\mathrm{SO}}_{3}^{-}}}$$

The definition of water flux is the mass of water passing through the membrane per unit area and time. The water flux is derived from the absolute humidity and the inlet air mass.

(2) $$\omega =\frac{{\dot{m}}_{v}}{{\dot{m}}_{a}}$$

(3) $${\dot{m}}_{v}=\omega \times {\dot{m}}_{a}=0.6219\frac{\phi P_{s}}{P_{a}}\times {\dot{m}}_{a}$$

The mass-conservation equation calculates the diffusion coefficient after taking the test jig with control volumes 1 and 2 separated by a Nafion^®^ membrane. C.V. 1 and 2 are dry and wet volumes of the test jig, respectively.

(4) $$C.V.1:\;{\dot{m}}_{i1,dry}+{\dot{m}}_{per,mem}={\dot{m}}_{o1,dy}+{\dot{m}}_{o1,vap}$$

(5) $$C.V.2:{\dot{m}}_{i2,wet}-{\dot{m}}_{per,mem}={\dot{m}}_{o2,dy}+{\dot{m}}_{o2,vap}$$

In this study, the diffusion coefficient was calculated under mass conservation of the membrane in terms of water content (λ).

(6) $$\begin{array}{l} N_{w}=-\frac{\rho_{dry}}{M_{mem}}D_{\lambda }\frac{\Delta \lambda }{\Delta z}. \end{array}$$

(7) $${\dot{m}}_{per,mem}=-\frac{\rho_{dry}}{M_{mem}}D_{\lambda }\frac{\Delta \lambda }{\Delta z}A$$

Because the mass transport through the membrane is calculated by Equations (4) and (5), and the water content is also calculated by the humidity of the wet and dry control volumes, the diffusion coefficient in Equation (7) can be determined.

## 3. Results and Discussion

### 3.1. Water Content of Nafion Membranes with Varying Temperature and Humidity

**Figure 5:**
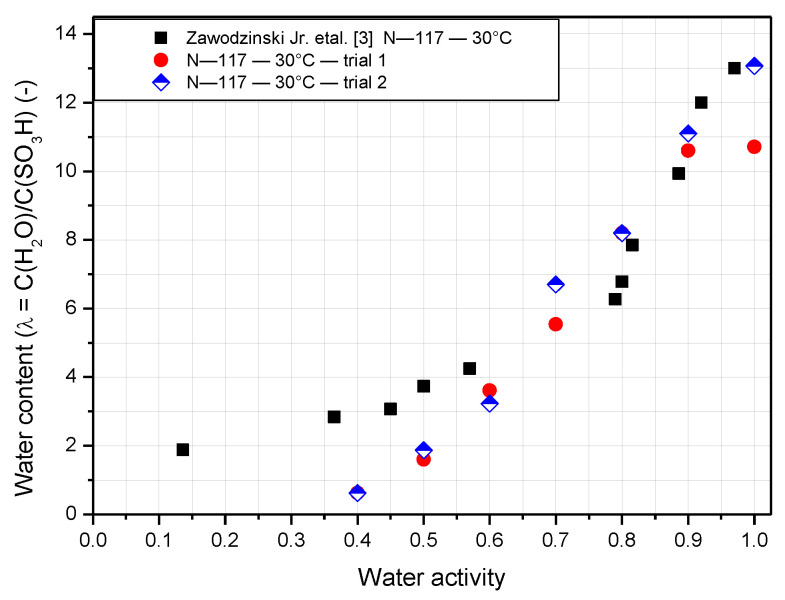
Comparison of water content of Nafion 117 membrane at an operating temperature of 30 °C.

**Figure 6:**
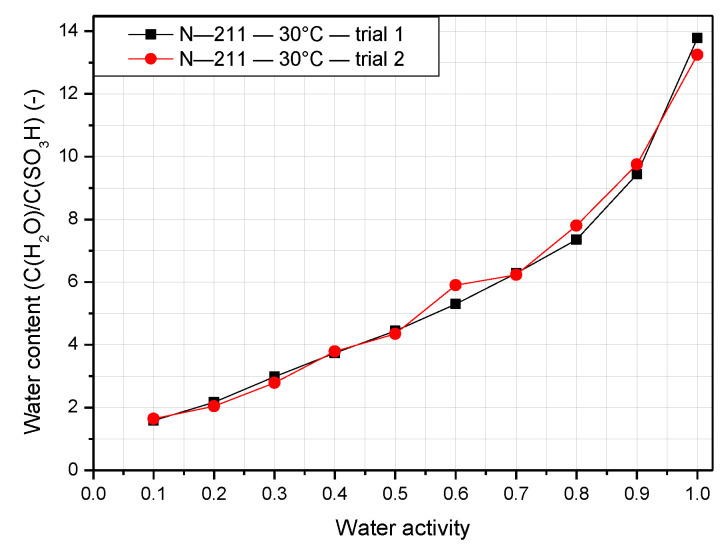
Water contents of Nafion 211 membrane at an operating temperature of 30 °C.

As mentioned previously, insufficient or excess water inside the stack results in a dramatic decline in performance and durability. Therefore, the water content in the membrane is an essential property that determines the performance of the proton exchange membrane (PEM). The water content of the Nafion membrane behaves differently depending on the operating temperature and relative humidity. Both N-117 and N-211 have an operating temperature of 30 °C, and N-117 is also tested at 50 °C and 70 °C, so the results can be compared clearly. Figure 5 shows the experimentally measured water content compared with the result of Zawodzinski [3]. The relative humidity is defined as the percentage of water activity, as indicated by the x axis of Figure 5. It is clear from the figure that when the gradient of water content increases slowly at low water activity, it is then accelerated at very high activity. This trend is observed both experimentally and in the literature. This is also observed for N-211, as indicated in Figure 6. Although the two trials show some deviations, there is only a slight difference between the results of N-117 found here and in the literature. Owing to experimental limitations, it is impossible to detect the water content for N-117 from Figure 5 when the water activity is less than 0.4. However, N-211 performs better in these conditions. The value of the water content exceeds 1.6 at a water activity of 0.1 and nearly reaches 3 at a water activity of 0.3. Additionally, the water content of N-211 is higher than that of N-117 at most points. The reason for that is because the method of fabrication of the N-211 is vastly different compared to N-117. As a result, N-211 appears to have larger ionic domains, which influence its water absorption. At a water activity of 1, the water content peaks at 13 for N-117 and nearly 14 for N-211. Furthermore, the value increases significantly as the water activity increases from 0.8 to 1.

**Figure 7:**
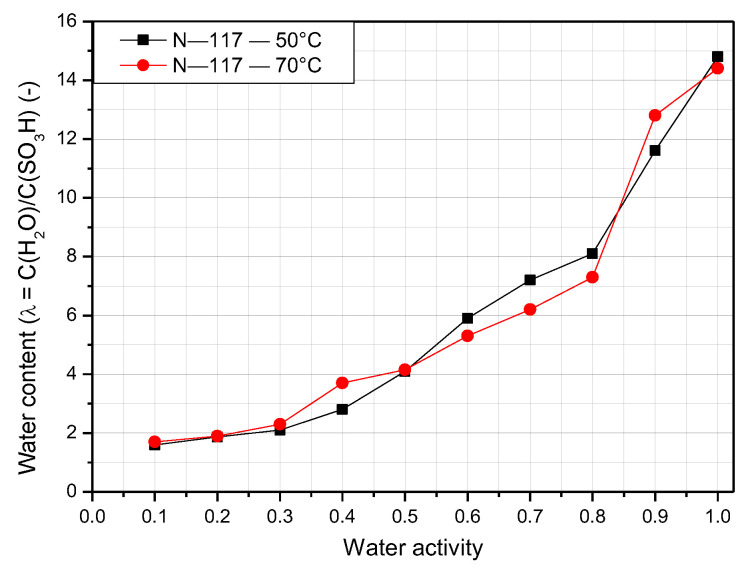
Water content of Nafion 117 membrane at operating temperatures of 50 °C and 70 °C.

Because the water content is defined by the concentration of water vapor to the concentration of sulfonic acid (fixed charge) inside the membrane, the water content seems to vary at high temperatures. Figure 7 shows the water content of the N-117 membrane at 50 °C and 70 °C at varying relative humidity. When the temperature increases, the water content tends to rise as well. The maximum value of λ at 50 °C is 15, and 70 °C s 14.9 at 100% relative humidity. The water content increases sharply when the temperature increases from 30 °C to 50 °C. However, there is a slight difference in water content between the temperatures of 50 °C and 70 °C. At higher temperatures, the water content of N-117 is detected easily when the water activity is low.

### 3.2. Water-Transport and Water-Diffusion Coefficient through Nafion Membrane

This section discusses the results of the effects of relative humidity and temperature on the water-transport and diffusion coefficient. The temperature is maintained on both sides of the test jig to achieve isothermal conditions in the experimental system. The pressure was also set the same on both sides to protect the structural configuration of the polymer when the system was tested at high temperature and relative humidity. As a result, the concentration gradient between the dry and wet sides is the only driving force, which means that the water-transport process is driven by the difference in vapor pressure between the feed water vapor on the wet side and the low partial pressure of the dry side. When the temperature of a mixture increases, the partial pressure rises simultaneously. Thus, the temperature is a crucial parameter affecting the system’s water-transport rate. The membrane is a medium for mass transfer between the wet and dry sides. Because the air is preprocessed through a compressor, drier, and filter before entering the system, the relative humidity on the dry side is sustained at a small percentage, and the measurement errors could be neglected. In other words, the air concentration on the dry side is stable in most experimental conditions. Therefore, the water-transport rate heavily depends on the water vapor concentration on the wet side. The relative humidity of the wet air flow is controlled from 10% to 100%.

**Figure 8:**
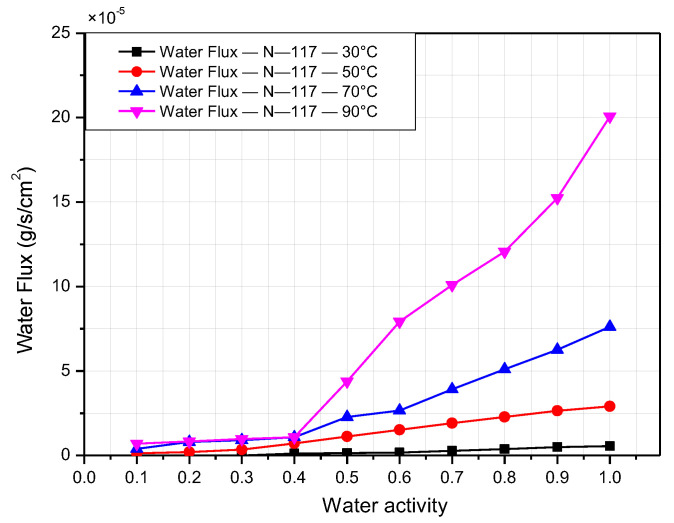
Nafion 117 membrane water flux according to relative humidity at operating temperatures from 30 °C to 90 °C.

**Figure 9:**
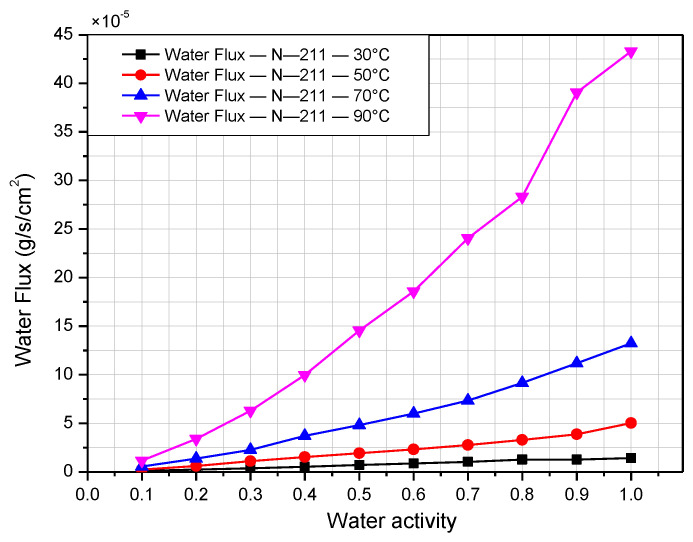
Nafion 211 membrane water flux according to relative humidity at operating temperatures from 30 °C to 90 °C.

Figure 8 and Figure 9 present the water flux of Nafion 117 and Nafion 211, respectively, according to the relative humidity at operating temperature from 30 °C to 90 °C. As demonstrated in the figures, when the relative humidity and temperature increase, the water flux rises simultaneously. Both graphs show the value of water flux on the order of 10^−5^ g/cm^2^ s. There is a slight difference in the water-transport rate for low water activity. However, the water-transport rate accelerates when the relative humidity is high. This is indicated clearly in the case of N-117. When the relative humidity is less than 40%, it is difficult to detect the difference in the water-transport rate at different operating temperatures. However, when the relative humidity exceeds 40%, the water flux increases considerably with increasing temperatures. In addition, the water-transport rate increases significantly when the temperature rises. This is explained by the exponential correlations between vapor pressure and temperature described by Antoine [20]. The highest difference is observed for the case between 30 °C and 90 °C. Under the same relative humidity, the water flux at 90 °C is almost ten times higher than that at 30 °C. The water-transport rate reaches its peak when the temperature is 90 °C and 100% humidity, approximately 20 × 10^−5^ g/cm^2^ s for N-117 and 41 × 10^−5^ g/cm^2^ s for N-211. According to the equation expressed by Springer [21] (Equation (6)), if λ, M, and D are all constant, then the water flux is proportional to the water content and membrane thickness. Moreover, the water-transport rate of N-211 more than double that of N-117 in most cases of temperature and humidity. This is because the water content of N-211 is higher than that of N-117 at the same temperature and relative humidity. In addition, the N-211 membrane is much thinner than the N-117 membrane.

**Figure 10:**
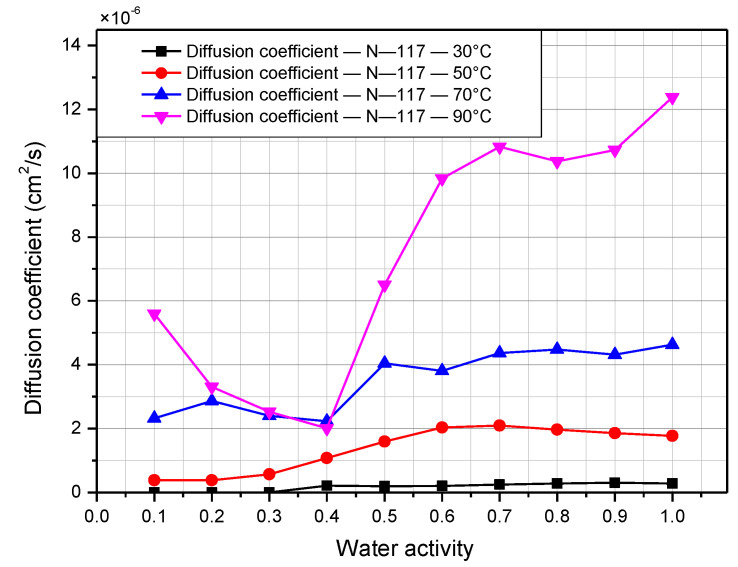
Diffusion coefficient of Nafion 117 membrane according to relative humidity at operating temperatures from 30 °C to 90 °C.

**Figure 11:**
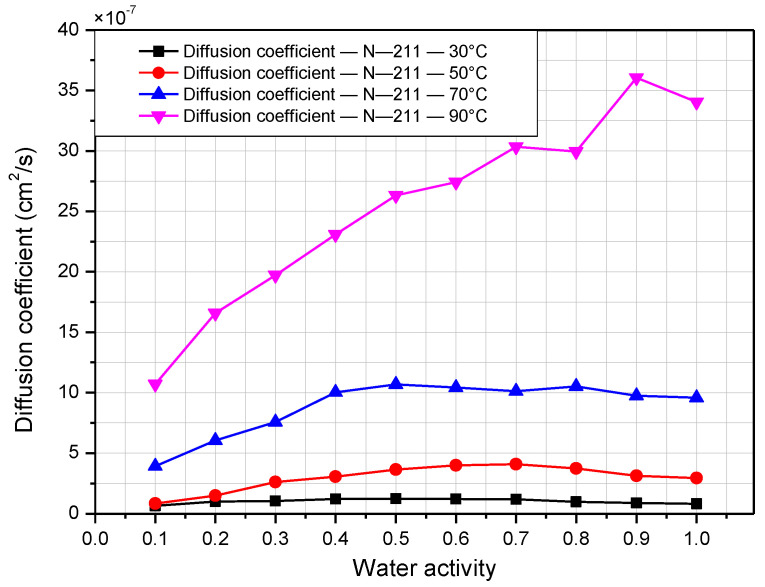
Diffusion coefficient of Nafion 211 membrane according to relative humidity at operating temperatures from 30 °C to 90 °C.

The diffusion coefficient is a crucial property that determines how quickly water can diffuse through the membrane, and depends on the surface concentration of the Nafion membrane. Temperature and relative humidity have significant impacts on this property. Figure 10 and Figure 11 presents the effect of temperature and humidity on the diffusion coefficient for Nafion 117 and 211. The value for the diffusion coefficient at 30 °C ranges from 0.11 × 10^−6^ to 0.54 × 10^−^^6^ cm^2^/s for N-117, which agrees well with data presented by G. Suresh [22]. K. Chadha reported diffusion coefficient values in the range from 1 × 10^−^^7^ to 4 × 10^−^^7^ cm^2^/s at a temperature of 60 °C for N-211 [19]. The value of the diffusion coefficient for N-211 in this work is quite similar to that, ranging from 0.4 × 10^−^^7^ to 2.6 × 10^−^^7^ cm^2^/s at 50 °C. The diffusion coefficient is generally proportional to the temperature and relative humidity. As the temperature and relative humidity increase, the diffusion coefficient also rises. The diffusion coefficient of N-117 is much higher than that of N-211, with values on the order of 10^−^^6^ and 10^−^^7^, respectively. The maximum value is over 12 × 10^−^^6^ cm^2^/s for N-117 at 90 °C and 100%, and 35 × 10^−^^7^ for N-211 at 90 °C and 90%.

## 4. Conclusions

An experimental study was carried out to compare water-transport properties of the electrolytic membranes Nafion 211 and Nafion 117. Some conclusions are as follows:

1. At the same temperature and humidity conditions, higher water content is absorbed by N-211 than N-117. Experimental limitations hindered the measurement of the water content of N-117 at relative humidity from 10% to 40%. Furthermore, as the temperature and relative humidity increase, the water content rises simultaneously.

2. There is a slight difference in the water-transport rate for low water activity. However, the water-transport rate accelerates when the relative humidity is at a high regime. The water-transport rate also increases significantly when the temperature rises for both types of membrane. The water-transport rate reaches its peak when the temperature is 90 °C and 100% humidity.

3. The diffusion coefficient is generally proportional to the temperature and relative humidity. Although the water-transport of N-211 is higher than that of N-117, the diffusion coefficient of N-117 is much greater than that of N-211.

## Figures and Tables

**Table 1:** Specification of the Nafion 117 and Nafion 211 membrane used in the experiment.

Parameter	Nafion 211	Nafion 117	Unit
Surface area	4900	4900	mm^2^
Thickness	25.4	183	µm
Width	7	7	cm
Length	7	7	cm
Equivalent weight	1100	1100	g/mol
Density	1.97	0.2	g/cm^3^

**Table 2:** Specifications of the components in the system.

Instrument	Model	Range	Accuracy
Mass flow controller	Line tech, Korea	100 L/min	±1%
Pressure sensor	IS-3, Wika	10 bar	±0.5%
Temperature controller	UT-32A, Yokogawa	−270–1370 °C	
Thermo-couple	K-type	−100–650 °C	±0.1 °C
Electronic scale	GX-200	0.001–210 g	±0.001 g
Vacuum dryer	OV-11, Jeiotech	25–250 °C	

**Table 3:** Experimental conditions for water-transport through the membrane.

Operating Variables	Value	Units
Flow rate	8	L/min
Temperature	30, 50, 70, 90	°C
Pressure	1	bar
Relative humidity	10–100	%
Flow arrangement	Co-flow	

## Data Availability

Not applicable.

## Nomenclature

λ	Water content
C.V.	Control volume of test Jig
$C_{H_{2}O}$	H_2_O molar mass, kg/kmol
$C_{SO_{3}^{-}}$	SO_3_—molar mass, kg/kmol
${\dot{m}}_{v}$	Vapor mass flow, kg/s
${\dot{m}}_{a}$	Air mass flow, kg/s
ϕ	Relative humidity
$P_{s}$	Saturation pressure, kPa
$P_{a}$	Air pressure, kPa
${\dot{m}}_{i1,dry}$	C.V. 1 inlet dry air, kg/s
${\dot{m}}_{per,mem}$	Water permeation through the membrane, kg/s
${\dot{m}}_{o1,dry}$	C.V. 1 outlet dry air, kg/s
${\dot{m}}_{o1,vap}$	C.V. 1 outlet vapor, kg/s
${\dot{m}}_{i2,wet}$	C.V. 2 inlet wet air, kg/s
${\dot{m}}_{o2,dry}$	C.V. 2 outlet dry air, kg/s
${\dot{m}}_{o2,vap}$	C.V. 2 outlet vapor, kg/s
$N_{w,diff}$	Molar flux, mol/cm^2^ s
$\rho_{dry}$	Membrane dry density, g/cm^3^
$M_{mem}$	Equivalent weight of the membrane, g/mol
D	Diffusion coefficient, cm^2^/s
ΔZ	Membrane thickness, cm
A	Membrane area, cm^2^
${\dot{m}}_{per,mem}^{"}$	Water flux through the membrane, kg/cm^2^ s
